# Influence of food emulsifiers on cellular function and inflammation, a preliminary study

**DOI:** 10.3389/fnut.2023.1197686

**Published:** 2023-08-02

**Authors:** Beatrice Dufrusine, Chiara Di Lisio, Andrea Maurizio, Michele Sallese, Vincenzo De Laurenzi, Enrico Dainese

**Affiliations:** ^1^Department of Bioscience and Technology for Food Agriculture and Environment, University of Teramo, Teramo, Italy; ^2^New Alimenta S.p.a., Pescara, Italy; ^3^Department of Innovative Technologies in Medicine and Dentistry, University “G. d’Annunzio” of Chieti-Pescara, Chieti, Italy; ^4^Center for Advanced Studies and Technology (CAST), University “G. d’Annunzio” of Chieti-Pescara, Chieti, Italy

**Keywords:** food emulsifiers, extra virgin olive oil, Caco-2 cells, macrophages activation, cytokines

## Abstract

Emulsifiers are extensively used as food additives and their consumption is increasing in Western countries. However, so far only few studies examined their potential effects on intestinal cellular functions and gut inflammation. The aim of this preliminary analysis was to study the emulsifiers and their concentrations capable of causing cellular damage compared to extra virgin olive oil (EVOO). We tested two commonly used emulsifiers (EMI, EMII) and EVOO on Caco-2 cells, derived from a colon carcinoma and widely used as a model of the intestinal inflammation. The diphenyltetrazolium bromide test MTT and clonogenic assay were used to study the effect of emulsifiers on cell viability. Cell migration was determined by the wound-healing assay. The inflammation was studied by measuring the levels of interleukin 6 (IL-6) and monocyte chemoattractant protein-1/C–C motif chemokine ligand 2 (CCL2), multifunctional cytokines with a major role in the acute-phase response. Furthermore, we analyzed the effect of conditioned media of Caco-2 cells treated with EMs on macrophages activation. In conclusion, our preliminary data provide evidence that EMs increase the proliferation and migration rate of Caco-2 cells. Moreover, Caco-2 cells treated with EMs enhance the IL-6 and CCL2 release and activated macrophages, supporting their role as proinflammatory molecules.

## Introduction

Emulsifiers are detergents extensively used as food additives, in particular in processed high fat-containing food, to stabilise the consistency of products, to prevent an unappetising separation of oil and water phases during storage, and as a vegetarian alternative to gelatine addition (1, 2). Consumption of highly processed foods are increasing in Western countries and a common feature of these food products is the use of at least one emulsifier (3, 4). Permitted emulsifiers commonly added to food are considered safe because they are probably largely degraded during the digestive process, mostly not absorbed, and therefore eliminated in feces (2, 5). So far there has been relatively little investigation on understanding the potential effects of dietary emulsifiers in intestinal permeability, in inflammation and in the pathogenesis of intestine diseases (6, 7). Previous research reported adverse effects of some approved and widely used food additives suggesting a link between these compounds, gut inflammation and allergic/intolerance reactions to food (6, 7). In particular, research has mainly focused on the side effects of the food additives carrageenan (CGN, E-407) for its capability to induce intestinal inflammation and ulceration *in vitro* and *in vivo* models (6–8). Several studies investigated the adverse effects of commonly used emulsifiers, such as carboxymethylcellulose (CMC) and polysorbate 80 (P80), on microbiome composition (9–12). These dietary emulsifiers induce a moderate disturbance of microbiota composition in animal models that is associated with continuous low-grade inflammation and metabolic disease (9, 13). The detergent-like properties of dietary emulsifiers might cause an alteration of the intestinal mucosal layers responsible for the gut protection from microbiota, thus increasing bacterial translocation across epithelia (9, 12, 14). The mucosal barrier is the primary physical defence and the disruption of the mucus–bacterial interactions can promote gut inflammation and immune response (15, 16). Furthermore, altered gut microbiota formation and/or composition is associated with numerous chronic inflammatory diseases, such as inflammatory bowel disease (IBD), obesity and metabolic syndrome (17, 18). The gastrointestinal tract (GI) is the body district with the largest number of macrophages, mostly in the *lamina propria* (19, 20). Gut resident macrophages interact with others immune cells and microbiota to modulate the mucosal immunity in various ways. Indeed, GI macrophages control the tolerance to food antigens, the turn-over of enterocytes by the uptake of apoptotic cells, and counteract inflammatory responses to microbial products (19). Extensive and prolonged immune response to environmental factors and intestinal microorganisms has been reported as a pathogenetic mechanism of IBD, Crohn’s disease (CD), and ulcerative colitis (UC) (21). Both CD and UC are characterized by chronic and superficial inflammation of the mucosa and submucosa in the GI tract (22). Epidemiological and nutritional trials supported a pivotal role for diet regarding inflammation in IBD and GI cancers, thus indicating nutrition as the most easily modulable risk factor to prevent and treat IBD and GI cancers (23, 24). The metabolic benefits of intake of extra-virgin olive oil (EVOO) have been reported in several animal and human studies (25–27). The components of EVOO, such as mono- and polyunsaturated fatty acids (MUFAs and PUFAs), saturated fatty acids, and acids phenolic compounds can exert biological activities due to their antioxidant, anti-inflammatory, and anti-proliferative properties (25–27). Indeed, the current trend in the food industry is to find sustainable production for new natural compounds, including EVOO, in alternative to synthetic food additives and emulsifiers (28, 29).

The mechanisms by which dietary emulsifiers can trigger acute and chronic inflammatory diseases are not fully understood, and previous studies were mostly focused on the influence on gut dysbiosis (10, 11). The aim of this study is to evaluate the putative effects of two commonly used dietary emulsifiers (EMI and EMII) on human colon adenocarcinoma Caco-2 cells proliferation, migration, and inflammation. We then examined the inflammatory effects of conditioned medium collected from EMs-treated Caco-2 (CM-EM) on THP-1 macrophages.

## Materials and methods

### Caco-2 cells culture and treatments

Caco-2 cells were obtained from ATCC (Rockville, MD, United States) and were routinely grown in Eagle’s Minimum Essential Medium (EMEM) supplemented with 10% FBS, 2 mM glutamine, penicillin (100 U/mL) and streptomycin (100 mg/mL) at 37°C in 5% CO2. Afterwards, Caco-2 cells were treated in serum-free medium with EMs at indicated concentrations using EVOO as vehicle for 24 h. We proceed with dose–response studies at this time based on our previously data of phenolic compounds on the same cell type (30). Thereafter, the supernatants (conditioned media, CM) were collected, centrifugated at 3000 × g for 10 min at 4°C, filtered and stored at −20°C until usage. For our analyses we chose two emulsifiers, that we called EMI and EMII, commonly used for chocolate spread and as Cocoa Butter Replacers (CBR) in confectionery products. They are all-vegetables and trans-free mixtures of fully saturated triglycerides. Their main characteristic is the formation of fine, stable crystals and their special oil-binding properties, which is why we chose EVOO as the vehicle for cell treatments. Furthermore, we reported no cellular effects on proliferation and inflammation due EVOO exposure at the dose and time used (Supplementary Figure S1).

### THP-1 macrophages culture and functional analysis

The human THP-1 cells were maintained in RPMI 1640 medium supplemented with 10% FBS and 1 mM sodium pyruvate (Gibco; Thermo Fisher Scientific, Inc.). We induced the differentiation of monocytes to macrophages as previously described (31). THP-1-derived macrophages were treated with the conditioned medium (CM) diluted 1:1 with serum free RPMI medium for 24 h. Thereafter, the supernatants were removed and after 24 h the supernatants were collected, centrifugated at 3000 g for 10 min at 4°C, filtered using a 0.2 μm Ministart sterile filter (Sartorius), and stored at −20°C until usage.

### MTT assays

For proliferation MTT assay, Caco-2 cells were seeded into 24-well plates at a density of 5 × 10^3^ cells/well in 500 μL of complete medium; they were treated with increasing doses of EMs or EVOO (ranging between 5 and 15 μg/mL) and further incubated for 24 h. At the end of the incubation period, cells were incubated with 200 μL of MTT solution (medium serum-free with 0.5 mg/mL of MTT) for a further 2 h. After removal of the MTT solution, 200 μL of dimethyl sulfoxide (DMSO) was added to the wells for 10 min, and the absorption value at 570 nm was measured using a multi-plate reader. All experiments were performed in triplicate.

### Colony formation assay

Caco-2 were treated with EMs at 10 μg/mL or EVOO for 24 h. At the end of treatments, 500 cells were plated on six-well plate in complete medium. After 14 days from the seeding, colonies were fixed and stained with a solution containing 1% Crystal Violet (Sigma-Aldrich). Images of the colonies were taken with EVOS M5000 microscope (Thermo Fisher Scientific). The number of colonies were obtained using ImageJ software (software version 1.50i, nih.gov, Bethesda, MD, United States).

### Wound healing assay

Caco-2 were treated with EMs at 10 μg/mL or EVOO for 24 h. At the end of treatments, were seeded on a six-well culture plate in complete growth medium. After 24 h, the cell monolayer was scratched with a sterile 200 μL pipette tip. Then, Caco-2 cells were washed twice with PBS and complete was added. After performing the scratch (T0) and at 24 and 48 h after treatment, images of the scratch area were taken with EVOS M5000 microscope (Thermo Fisher Scientific). The scratch area was measured using ImageJ software (software version 1.50i, nih.gov, Bethesda, MD, United States) and normalized to time T0 scratch. Two independent experiments were performed. Results are shown as mean ± standard deviation (SD). The multiple unpaired t test was chosen for analysis between two groups at different times using GraphPad Prism 9 (GraphPad Software, San Diego, CA, United States). The significance level was set at *p* < 0.05.

### ELISA

The concentration of IL-6 in the cellular supernatants were determined using the Human IL-6 Uncoated and CCL2 purchased by Invitrogen ELISA Kit assay (ThermoFisher, San Diego, USA) applying the manufacturer’s directions. The plates were read at 450 nm and the sensitivity of the used ELISA assay was in the range 2–200 pg./mL for IL-6 and 7–1,000 pg./mL for CCL2.

### Statistical analysis

For analyzed data, *p* values were determined by Mann–Whitney test and considered significant for *p < 0.05, **p < 0.005, and ***p < 0.005. Experimental sample numbers (n) are indicated in the figure legends. All statistical analysis was performed with GraphPad Prism 8.0 and SPSS 15.0 software.

## Results

### Food emulsifiers enhance Caco-2 cells proliferation

**Figure 1:**
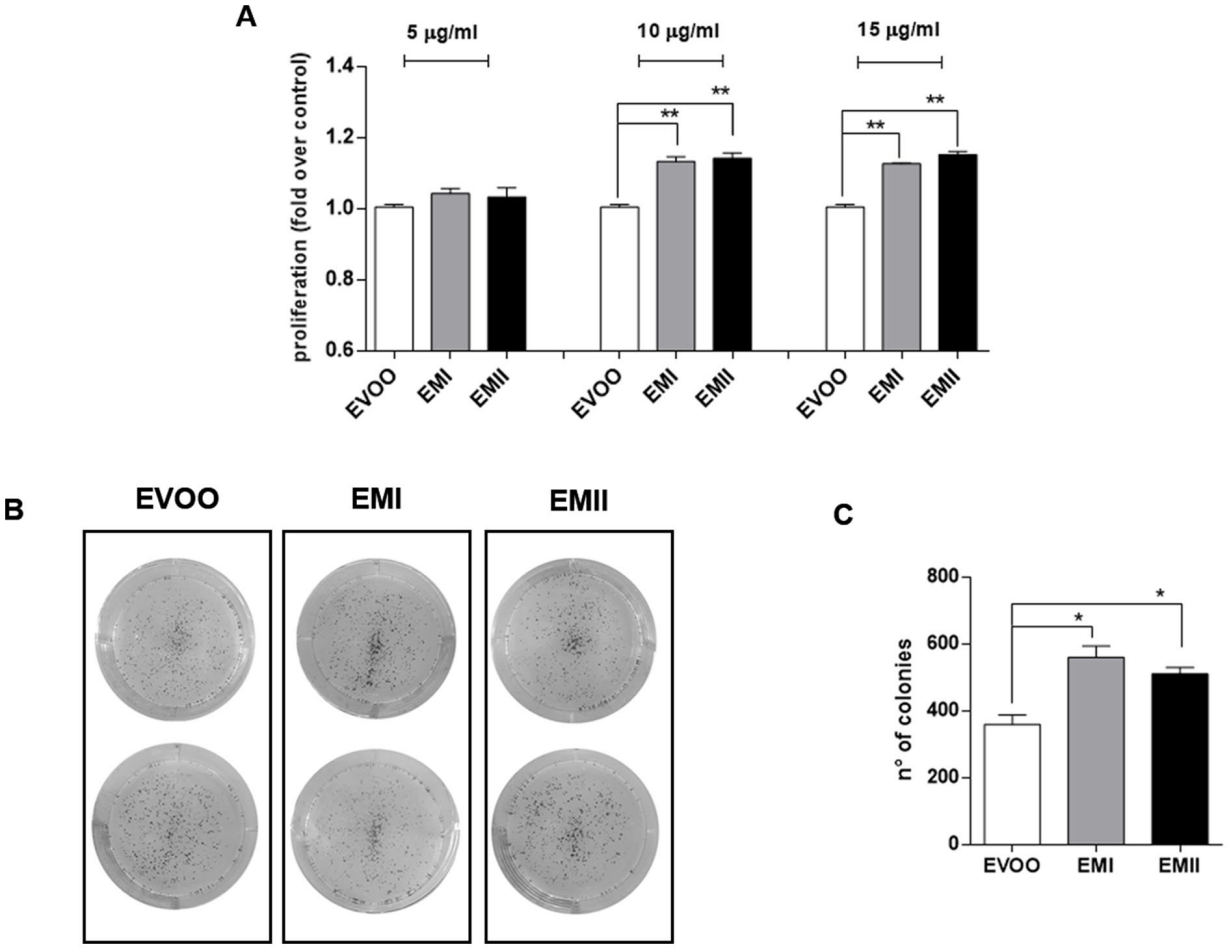
**(A)** Emulsifiers increase Caco-2 cell proliferation. The effect of EMI, EMII and EVOO vehicle on cellular proliferation was obtained by using the MTT assay. Caco-2 cells were treated for 24 h with food emulsifiers at the concentrations of 5. 10 and 15 μg/mL for 24 h. **(B)** Representative image of the clonogenic assay. **(C)** Analysis of colony-forming efficacy of Caco-2 cells after exposure to EMs and EVOO vehicle at 10 μg/mL for 24 h. Data are expressed as number of colonies calculated as percentage compared to EVOO, using imageJ software. The data values are presented as means of three and two experiments ± SD, which expressed using for control cells grown in medium with EVOO.

The proliferative effect of food emulsifiers on Caco-2 cells was investigated by MTT and clonogenic assays. Caco-2 cells were treated with increasing emulsifiers concentration (from 5 μg/mL to 15 μg/mL) for 24 h. EMs treatments caused a significant and dose dependent increase of Caco-2 cell line proliferation by MTT analyses (Figure 1A). Specifically, EMs treatments at 10 and 15 μg/mL induced a significantly increase in the proliferation rate compared to EVOO vehicle-treated cells (*p* = 0.007 EMI at 10 μg/mL, *p* = 0.007 EMII at 10 μg/mL, *p* = 0.001 EMI at 15 μg/mL and *p* = 0.003 EMII at 15 μg/mL) (Figure 1A). Furthermore, we performed clonogenic assay to evaluate the food emulsifiers long-term effect on cell growth. As reported in Figures 1B,C, clonogenic assay showed similar effects of MTT assay. The emulsifiers increased the capacity of Caco-2 to form colonies compared to EVOO (*p* = 0.045 EMI vs. EVOO, *p* = 0.047 EMII vs. EVOO) (Figures 1B,C). In particular, EMs treatments increased not only the colonies number but also the colonies size compared to EVOO (Figure 1B).

### Effects of food emulsifiers on cellular migration by wound healing test

**Figure 2:**
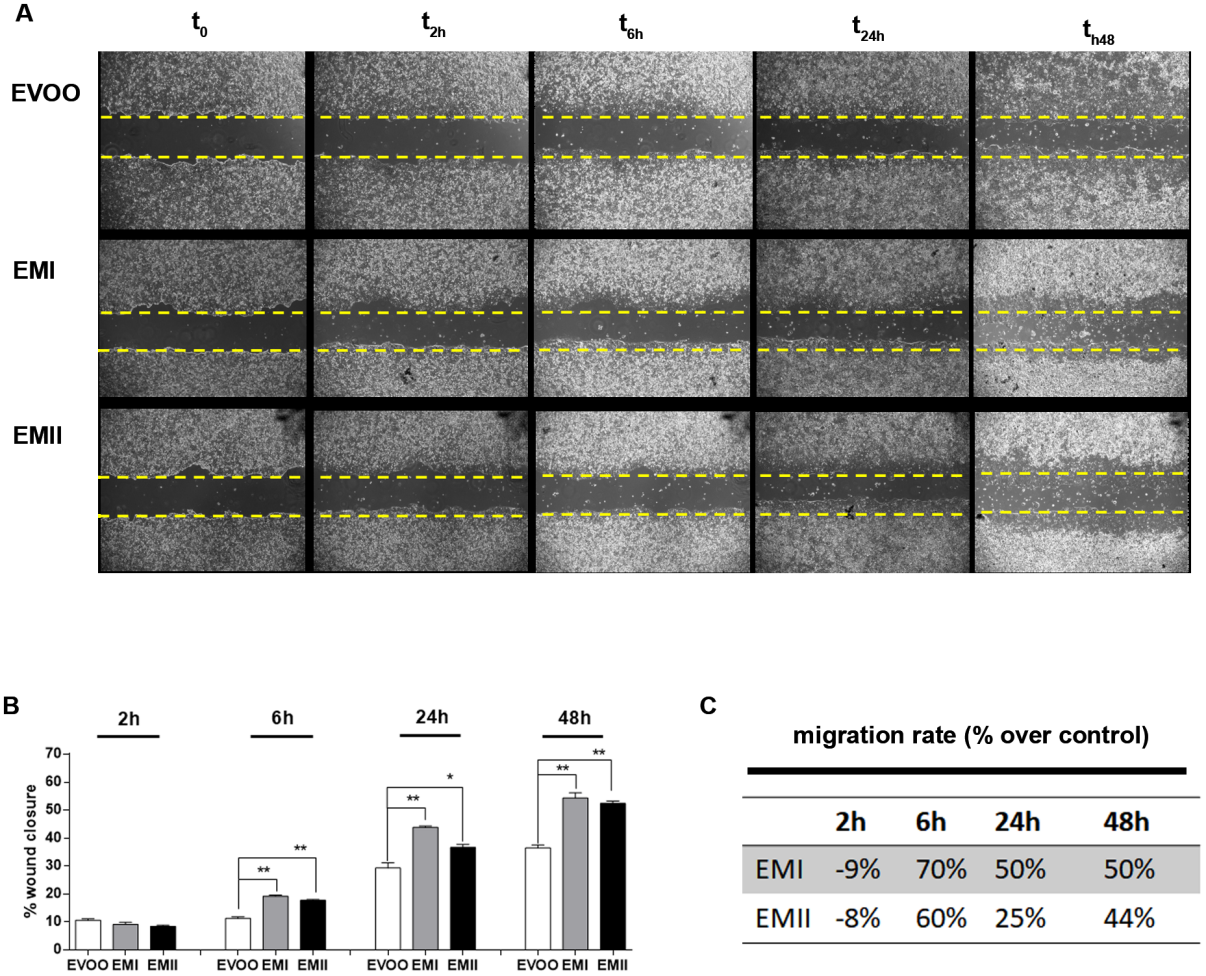
Effect of EMs on migration of Caco-2 cells. **(A)** The representative images of wound site in Caco-2 treated with emulsifiers at 10 μg/mL at time 0, 2, 6, 24 and after 48 h. **(B,C)** The gap width was measured and wound healing rates and time-scales for EMs compared to EVOO and have been reported. The data are represented as mean of three experiments ± SD of triplicate experiments.

The wound healing assay (Figure 2A) showed a significant increase of cell migration rate in the scratched area following incubation of Caco-2 cells with EMI and EMII compared to the EVOO control condition. Following EMs treatments at 10 μg/mL for 24 h, a significant increase of percentage of cell migration rate occurred in the scratched area versus EVOO group (+70%, +50%, and + 50 for EMI and + 60%, +25%, and 44% for EMII, respectively vs. EVOO group) (Figures 2B,C).

### Effects of food emulsifiers on IL-6 and CCL2 secretion by Caco-2 cells and THP-1-derived macrophages

**Figure 3:**
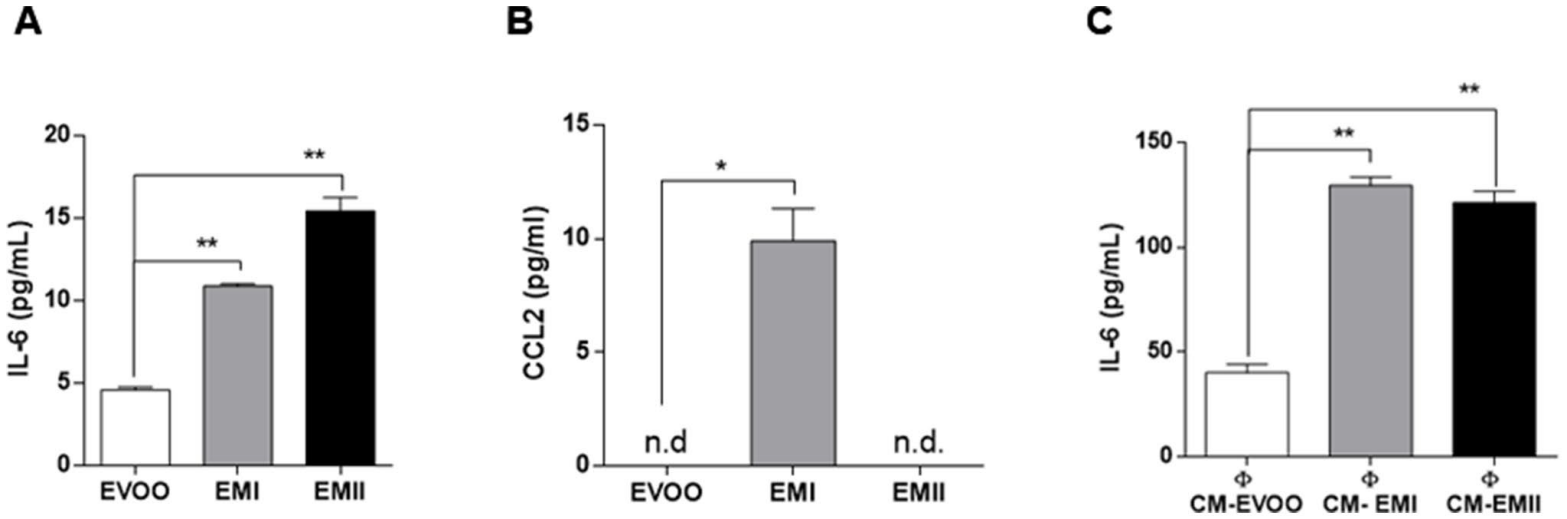
Direct and indirect immunomodulatory effect of EMs. Production of IL-6 **(A)** and CCL2 **(B)** in Caco-2 cells in response to EMs treatments. Caco-2 cells were treated with EMs and EVOO vehicle for 24 h and IL-6 and CCL2 levels were detected on supernatants by ELISA. Data showed that both EMs increased significantly IL-6 levels and only EMI treatments induced CCL2 release. **(C)** Effect of CM-EM and CM-EVOO on IL-6 levels released from THP-1 macrophages was evaluated by ELISA. The IL-6 levels in supernatants were analyzed after 24 h treatment through ELISA assay.

In order to elucidate the inflammatory effect observed by food emulsifiers treatments we measure IL-6 and CCL2 levels, by ELISA. Caco-2 cells treated with EMI and EMII, had significantly higher expression levels of IL-6 with respect to EVOO vehicle-treated cells. Results showed that the inflammatory cytokine IL-6 was produced significantly (*p* = 0.009 EMI vs. EVOO and *p* = 0.008 EMII vs. EVOO) higher by Caco-2 cells treated with emulsifiers as compared to the cells exposed to EVOO vehicle (Figure 3A). These data are in accordance with the effect of EMs on migration of Caco-2 cells. Indeed the Caco-2 cells migration is common reported during the inflammatory response (32). Interestingly, our data shown that the CCL2 cytokine not released from Caco-2 cells (Supplementary Figure S1) is induced only from EMI treatments (Figure 3B). Furthermore, we analyzed the immunomodulatory effects of conditional media isolated from Caco-2 cell stimulated with EMI, EMII (CM-EM), and EVOO (CM-EVOO) on THP-1 macrophages. As presented in Figure 3C, CM-EM significantly increased the release of IL-6, compared with the CM-EVOO group (*p* = 0.017 EMI vs. EVOO and *p* = 0.047 EMII vs. EVOO). The results showed that EMs can induce a direct inflammatory response on Caco-2 cells and also an indirect inflammatory response on immune cells.

## Discussion

Western dietary habits are characterized by increased intake of processed foods and consequently food additives. Food additives enhance shelf life and taste of processed foods and include emulsifiers. Emulsifiers allow homogenization of immiscible liquids and are added into many processed foods (1, 2). The emulsifiers effect on disturbance of microbial composition promoting susceptibility to chronical intestinal inflammation, has been reported in animal and human studies (10, 11). Acute and chronic gut inflammation promotes the development of intestinal inflammatory disorders, such as IBD and metabolic syndrome (22, 33). The incidence and prevalence of IBD and GI cancers is increasing in 21st century, in particular among Westernized countries (34, 35). Dietary changes in these countries, including increased intake of refined sugars and reduced intake of fibre, have been reported as risk factors for the development of IBD (36, 37). Patients with IBD have an increased risk of developing GI cancers due to the chronic inflammatory condition (33, 38). Recent studies reported that most of GI cancers are caused by modifiable risk factors, including alcohol consumption and smoking, as well as diet (24, 39, 40). Diet contributes to gut inflammation through modulation of the gut microbiota composition, alteration of intestinal mucosal permeability and antigen presentation. Furthermore, nutritional trials with exclusive enteral nutrition (EEN) restricting Western dietary habits effectively led to consider IBD as a metabolic syndrome (23, 41). Interestingly, dietary pattern characterized by olive oil, especially EVOO, was inversely associated with the GI inflammation (30, 42). Several animal studies reported the beneficial role of dietary EVOO in different experimental colitis models describing the antiatherogenic and the anti-inflammatory properties (30, 43–46). The aim of this preliminary study is to investigate the effect of food EMs on human colorectal adenocarcinoma cell line CaCo-2. Caco-2 cells possess the ability to produce various inflammatory cytokines so this cellular model are commonly used in inflammatory studies such as IBD and colon cancers (47). We tested two emulsifiers with oil-binding properties, so we decided to use EVOO as vehicle. Afterwards we demonstrated no cellular effect due to EVOO exposure at the dose and time we used as vehicle (Supplementary Figure S1); therefore we proceeded to compare EMs treatments with respect to EVOO group. Increased cellular proliferation and cell motility are hallmark of IBD and GI cancer (48, 49) so we tested the EMs effect on Caco-2 cell viability and migration. Here we demonstrated that increasing concentrations of EMs are accompanied by a significant increase in proliferation and migration rates in Caco-2 cells (Figures 1, 2). The enhanced proliferation and migration rate are characteristic of intestinal inflammatory lesions in IBD and promote colon cancer development, in particular for the colitis-associated colorectal cancers (50, 51). Previous studies reported that the inflammation of intestinal mucosa induces changes in the enterocytes to acquire an antiapoptotic and migratory phenotype, associated to carcinogenesis (52, 53). Furthermore, we reported that EMs can induce an increase in IL-6 release by Caco-2 cells (Figure 3A). It is well known that IL-6 control a plethora of effects during both acute and chronic inflammation, and its expression resulted modulated during IBD and GI cancers (38, 54). IL-6 is one of the inflammatory cytokines that directly or indirectly affect the intestinal epithelial cells, leading to the injury or necrosis of these cells, which promotes the development of IBD (38, 54). Furthermore, in IBD patients IL-6 increased at mucosal and systemic levels and its expression level correlates with the disease severity (54). When IBD occurs, inflammation increased the proliferation of the *lamina propria* cells mainly consisting of lymphocytes, and macrophages (33, 55). Although different cell types can contribute to increased IL-6 levels in the intestinal mucosa during inflammation, enterocytes and macrophages represented the main cell types responsible for the IL-6 secretion in IBD (55, 56). Our data showed that EMs act directly on Caco-2 cells inducing an increase of IL-6 levels (Figure 3A). Furthermore, we observed the effects of conditioned media recovered from Caco-2 cells EMs-treated (CM-EMs) on macrophages. We reported that Caco-2 cells stimulated from EMs CM-EMs can enhance the release of IL-6 from macrophages (Figure 3C), thus further contributing to the inflammatory state.

We also investigated the EMs effect on the release of CCL2 from Caco-2 cells. This cytokine, also known as monocyte chemotactic protein 1 for its chemoattractant properties, resulted up-regulated in Caco-2 cells following various inflammatory stimulus (57, 58) and its inhibition reduces inflammation in animal model for IBD (59, 60). Our data show an undetectable CCL2 basal level in Caco-2 (Supplementary Figure S1; Figure 3B) and an induction of this cytokine after EMI treatments (Figure 3B). These results are in line with the complex cytokines profile reported in IBD (61) and with previous studies describing a pro-inflammatory effect of the food additives, such as CGN, due to the increasing of cytokines and reactive oxygen species in cellular and animal models (62, 63).

These preliminary data support the concept that the increase of processed food consumption may be linked to the increase of IBD, and GI cancers. Indeed, EMs can induce an increase of migration and proliferation rate of colon cancer cells and cytokines release (Figures 1–3). Furthermore, Caco-2 cells EMs-stimulated can further increase the IL-6 levels by macrophages activation (Figure 3). The aim of future studies will be to investigate into the details of the molecular pathways activated in a distinctive or common way by the two emulsifiers leading to the release of inflammatory cytokines. Overall, these data suggest the need of a more careful and extensive research on all the EMs used in food to better assess their differential role in determining membrane modifications, alterations of specific proinflammatory pathways as well as their role in cancer.

## Data availability statement

The original contributions presented in the study are included in the article/Supplementary material, further inquiries can be directed to the corresponding author.

## Author contributions

BD and ED contributed to conception and design of the study. BD performed the experiments, interpreted data, and wrote the first draft of the manuscript. CL, AM, and MS read and corrected the paper. VL suggested ideas, read and wrote the paper. ED designed the study, supervised the project and wrote the paper. All authors contributed to the article and approved the submitted version.

## Funding

This work was supported by Department of Bioscience and Technology for Food Agriculture and Environment (VITALITY-project PNRR), University of Teramo.

## Conflict of interest

CL and AM are employed by New Alimenta S.p.a.

The authors declare that this study received funding from New Alimenta S.p.a. The funder was not involved in the study design, collection, analysis, interpretation of data, the writing of this article, but only in the reading and correcting the manuscript as well as in the decision to submit it for publication.

## Publisher’s note

All claims expressed in this article are solely those of the authors and do not necessarily represent those of their affiliated organizations, or those of the publisher, the editors and the reviewers. Any product that may be evaluated in this article, or claim that may be made by its manufacturer, is not guaranteed or endorsed by the publisher.
