# Supplementary material for: Influence of food emulsifiers on cellular function and inflammation, a preliminary study
Source: Front Nutr. 2023 Aug 2;10:1197686. doi: 10.3389/fnut.2023.1197686 (PMC10434242; doi:10.3389/fnut.2023.1197686)
Supplement: Supplementary file 1 [file Data_Sheet_1.docx]

Supplementary Material

Influence of food emulsifiers on cellular function and inflammation, a preliminary study

Beatrice Dufrusine^1^, Chiara Di Lisio^2^, Andrea Maurizio^2^, Michele Sallese^3,4^, Vincenzo De Laurenzi^3,4^ and Enrico Dainese^1*^

*** Correspondence:** Enrico Dainese: edainese@unite.it

# Supplementary Data

The emulsifiers that we used in this work and we called as EMI and EMII are commonly applied for chocolate spread and as cocoa Butter Replacers (CBR) in confectionery products. They are all-vegetables and trans-free mixture of fully saturated triglycerides. Their mainly characteristic is stable and fine crystal formation and special oil-binding properties, reason why we chose oil as our vehicle for cellular treatments. First of all, we measured eventually cellular effects due to EVOO vehicle exposure. As shown in Supplementary Figure 1, we reported no changes in proliferation, migration and cytokines release due to EVOO exposure at the dose and time used.


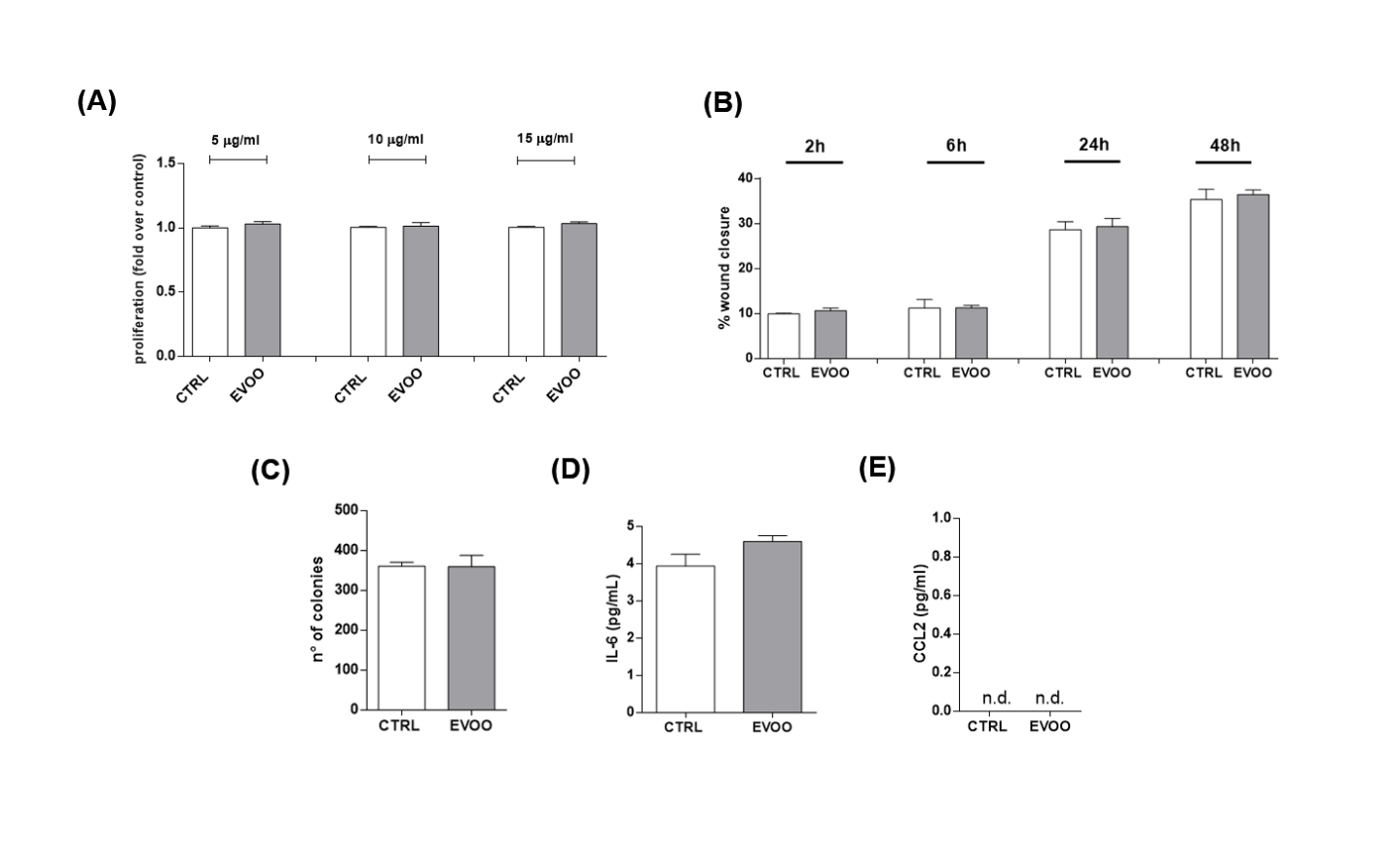


**Supplementary Figure 1.** **No cellular effects due to EVOO exposure as vehicle are evident in** **Caco-2 cell.** The effect of EVOO on cellular proliferation was obtained by using the MTT assay (A) and clonogenic assay (C). Caco-2 cells were treated for 24 h with food emulsifiers and EVOO at the concentrations of 5. 10 and 15 µg/ml for 24 h. (C) Analysis of colony-forming efficacy of Caco-2 cells after exposure to EMs and EVOO at 10 µg/ml for 24 h. Data are expressed as number of colonies calculated as percentage compared to EVOO, using imageJ software. The data values are presented as means of three and two experiments ± SD, which expressed using for control cells grown in medium with EVOO. (B) The wound healing assay reported not significant increase of cell migration rate in the scratched area following incubation of Caco-2 cells with EVOO. The gap width was measured and represented as mean of three experiments ± SD of triplicate experiments. Production of IL-6 (D) and CCL2 (E) in Caco-2 cells in response to EVOO exposure. Caco-2 cells were treated with EVOO for 24 hours and IL-6 and CCL2 levels were detected on supernatants by ELISA. Data showed that EVOO not induce changes in cytokines release compared to basal levels.
